# Microbubble-based enhancement of radiation effect: Role of cell membrane ceramide metabolism

**DOI:** 10.1371/journal.pone.0181951

**Published:** 2017-07-26

**Authors:** Azza Al-Mahrouki, Anoja Giles, Amr Hashim, Hyunjung Christina Kim, Ahmad El-Falou, Dean Rowe-Magnus, Golnaz Farhat, Gregory J. Czarnota

**Affiliations:** 1 Radiation Oncology and Physical Sciences, Sunnybrook Health Sciences Centre and Sunnybrook Research Institute, Toronto, Canada; 2 Departments of Radiation Oncology and Medical Biophysics, University of Toronto, Toronto, Canada; 3 Department of Biology, Indiana University, Bloomington, United States of America; Swedish Neuroscience Institute, UNITED STATES

## Abstract

Ultrasound (US) stimulated microbubbles (MB) is a new treatment approach that sensitizes cancer cells to radiation (XRT). The molecular pathways in this response remain unelucidated, however, previous data has supported a role for cell membrane-metabolism related pathways including an up regulation of UDP glycosyltransferase 8 (UGT8), which catalyzes the transfer of galactose to ceramide, a lipid that is associated with the induction of apoptotic signalling. In this study, the role of UGT8 in responses of prostate tumours to ultrasound-stimulated microbubble radiation enhancement therapy is investigated. Experiments were carried out with cells *in vitro* and tumours *in* vivo in which UGT8 levels had been up regulated or down regulated. Genetically modified PC3 cells were treated with XRT, US+MB, or a combination of XRT+US+MB. An increase in the immunolabelling of ceramide was observed in cells where UGT8 was down-regulated as opposed to cells where UGT8 was either not regulated or was up-regulated. Clonogenic assays have revealed a decreased level of cellular survival with the down-regulation of UGT8. Xenograft tumours generated from stably transfected PC3 cells were also treated with US+MB, XRT or US+MB+XRT. Histology demonstrated more cellular damage in tumours with down-regulated UGT8 in comparison with control tumours. In contrast, tumours with up-regulated UGT8 had less damage than control tumours. Power Doppler imaging indicated a reduction in the vascular index with UGT8 down-regulation and photoacoustic imaging revealed a reduction in oxygen saturation. This was contrary to when UGT8 was up regulated. The down regulation of UGT8 led to the accumulation of ceramide resulting in more cell death signalling and therefore, a greater enhancement of radiation effect when vascular disruption takes place through the use of ultrasound-stimulated microbubbles.

## Introduction

Tumour microvasculature is very important in radiation responses and it was recently shown that apoptotic death of microvascular endothelial cells is required for tumour cure [1, 2]. Exposing tumour vasculature to single large doses of radiation (>8–10 Gy) causes endothelial cell death, ceramide signalling was reported to be involved [3–5] Ceramide production is dependent in part on sphingomyelinases and is the favored biochemical mechanism leading to endothelial cell death due to the relative high levels of these enzymes. Tumour cell death is, thus, enhanced as a result of endothelial cell death leading to microvascular deterioration.

Several recent reports have suggested an enhancement of the radiation response using ultrasound-activated microbubbles [2, 3, 6–13]. These 1–8 μm diameter bubbles are composed of a gas core (usually nitrogen, air, or a perfluorocarbon) stabilized by a thin lipid or protein shell [14, 15]. Of particular interest, however, is that microbubbles can be stimulated when exposed to acoustic pressures at or near their resonant frequency. The resulting cavitation of the bubbles induces a reversible perforation of nearby endothelial cell membranes, allowing the passage of large molecules into the cells. This increased membrane permeability, known as sonoporation, has been demonstrated to enhance gene transfer and drug delivery [16–18]. Furthermore, microbubbles disruption by acoustic waves may lead to shockwaves and the formation of local micro jets that can destroy cellular membranes [19]. *In vivo* experiments have indicated that acoustic bubble stimulation combined with a single 2–8 Gy dose radiation, resulted in up to 60% tumour cell death within 24 hours of the single combined treatments [2, 6–13]. In those studies, several mouse tumour xenograft models were investigated including prostate (PC3), breast (MDA-MB-231) and bladder (HT-1376) cancers. Results indicated low levels of cell death with the administration of either a single 2Gy dose of radiation (4%–15% cell death) or a single ultrasound-activated microbubble treatment (10%– 15% cell death), while the single combined treatments resulted in significant cell death (25%–45%). These are all tumour types where radiotherapy can be used in the up-front treatment of disease and are accessible to focused ultrasound energy.

Changes in tumour vasculature were further assessed using high frequency power Doppler imaging before and after treatments in prostate [7], bladder [11] and breast [20] tumour xenograft models. Decreases of 2%–25% and 8%–30% in the vascular index were observed with single treatments of XRT and US+MB, respectively, while a significant drop in vascular index of 27%–52% was measured with the combined treatment [7, 11, 20]. Additionally, in a prostate (PC3) tumour model study using multiple combined treatments of XRT+US+MB, a dose of 2 Gy (BED10 = 38.4 Gy) combined with US/MB demonstrated increased survival and tumour regression when compared to a curative dose (BED10 = 58.5 Gy) of radiation alone [2].

Gene profiling was evaluated *in vitro* in HUVEC cells, a model for endothelial cells, indicating the up-regulation of genes coding for proteins responsible for apoptotic ceramide signalling and metabolism [3]. These included genes for sphingomyelinases, UDP glycosyltransferase 8 (UGT8), cytochrome C, and caspase 9. Apoptotic ceramide signalling was further evaluated both *in vitro* in HUVEC cells and *in vivo* in PC3 xenografts using exogenous ceramide and sphingosine-1-phosphate (S1P), a pro-survival signaling molecule that induces a contrasting effect to ceramide. Exposure to S1P resulted in diminished apoptotic cell death in response to US+MB+XRT combined treatments, suggesting an up-regulation of ceramide cell death signalling caused by US+MB therapy [2, 3, 21].

The mechanism of action for combined US+MB+XRT treatments involves a ceramide-related stress response in endothelial cells damage by microbubble stimulation. When combined with radiation treatment, endothelial cell death ensues, disrupting tumour vasculature and leading to tumour cell death. The exact factors responsible for modulating the link between endothelial cell damage and tumour cell death remain unclear. However, one candidate molecule is UGT8, which mediates the response of tumour cells to stress and is also involved in cell membrane repair.

The main function of UGT8 is galactosylceramide synthesis through the transfer of UDP-galactose to ceramide. This enzyme is localized to the nuclear envelope and the endoplasmic reticulum and has been reported to act as an anti-apoptotic molecule that affects tumorigenic and metastatic properties of breast cancer cells [22]. UGT8 is also linked to the development of lung metastases and is up-regulated in both ovarian and breast cancers [23–25]. Its anti-apoptotic function is achieved through disruption of the ceramide signalling pathway by converting ceramide into galactosylceramide.

The experiments conducted here probe the role of UGT8 in modulating the cell stress response to ultrasound-stimulated microbubble enhancement of radiation therapy responses. The study relies on an approach of generating a “loss of function” phenotype through the use of small inhibitory RNA targeting UGT8 messenger RNA in PC3 cells. The study uses wild-type PC3, and PC3 stably transfected with either scrambled nucleic acids (controls) or with the coding sequence of *UGT8* for up-regulation. The PC3 model was selected for the work here as a model system in which the fundamentals of ultrasound-stimulated microbubble enhancement of radiation effect have been established. Here we manipulated one genetic component (*UGT8*) which is involved in lipid biosynthesis, in order to probe the fundamental question of how ceramide related effects at the endothelial layer of blood vessels which cause vascular disruption, are translated into tumour parenchyma resulting in gross damage to tumour cells. This model system is relatively stable, predictable for growth, and one which could be genetically manipulated to increase and decrease UGT8 levels in a controlled manner.

Data were evaluated using histopathology and immunohistochemistry methods. In addition, functional approaches such as photoacoustic and power Doppler imaging are used to detect change in oxygen saturation and in blood flow. The results indicate an important role for UGT8 in affected responses of tumour cells to ultrasound stimulated microbubble radiation enhancement treatments.

## Materials and methods

### Cell culture

A prostate cancer cell line (PC3) was obtained from American Type Culture Collection, ATCC (CRL-1435; Manassas, VA, USA). Cells were cultured in RPMI-1640 media, obtained from Wisent biocentre (350-000-CL; St-Bruno, Quebec, Canada), and supplemented with 10% serum from Hyclone (KTC 30727; Thermo Fisher Scientific, Waltham, MA) and 100 U/mL of penicillin/streptomycin from Invitrogen (15140; Carlsbad, CA). Cells were grown and maintained under humidity and at 37°C, 5% CO2. Confluent cells were harvested using 0.05% trypsin-EDTA from Invitrogen (25300–062, Carlsbad, CA).

### Transient and stable transfection

A HuSH 29 mer shRNA construct against *UGT8* in pGFP-V-RS vector, and a GFP-tagged ORF clone of Homo sapiens *UGT8*, transcript variant 2 in pCMV6-AC-GFP vector, were obtained from OriGene (Rockville, MD, USA) and were used in transfection of PC3 cells. In addition, a scrambled negative shRNA control (also obtained from OriGene) was used as a control. The construct with the following sequence:

5’-TCACCTACGTGCCGCTGTCCATCAGATCT-3’ demonstrated about 50% down regulation of *UGT8* levels when tested using real time RT-PCR, was used in transfection for experiments. PC3 cells were plated one day before transfection. The DNA plasmid/turbofectin 8.0 (OriGene; Rockville, MD, USA) complexes were prepared and then incubated at room temperature for 45 min, after which they were added directly to cell cultures and were incubated in 5% CO2 incubator at 37°C. Cultured cells were subjected to antibiotic selection, 48 hours after transfection to generate stably transfected cells. The antibiotics that were used in the selection were puromycin dihydrochloride (used at a concentration of 0.5 μg/mL) with cells transfected with shRNA, and neomycin (G-418; Geneticin) used at a concentration of 200 μg/mL which was used with cells transfected with the open reading frame (ORF) of *UGT8*. Both antibiotics were obtained from Invitrogen (Carlsbad, CA. USA).

### Evaluation of UGT8 levels of expression

RNA was extracted from non-transfected and transfected PC3 cells using Qiagen RNeasy Mini Kit (Valencia, CA) according to the manufacturer’s instructions. *UGT8* expression levels were then evaluated using real-time RT-PCR which was carried out using a power SYBR green RNA-to-CT 1-step kit (Applied Biosystems, Foster City, CA).

The protocol was performed according to the manufacturer’s recommendations for quantification experiments on a real-time PCR system and data were analyzed as previously described [3] using the 2^-ΔΔ Ct^ method [26, 27] to assess the change in gene expression level relative to other samples [28].

In order to evaluate UGT8 protein levels, an enzyme-linked immunosorbent assay (ELISA) was used. Transfected cells were grown to confluence, collected, counted, and were then homogenized in PBS. Supernatants were then assayed with UGT8 detection carried out using an ELISA kit (Cat# MBS053427 from MyBiosource, San Diego, CA), following the manufacturer's instructions.

### Ceramide metabolism and trafficking in living cells

Stably transfected cells were rinsed with 1X Hank’s balanced salt solution (HBSS) and were then incubated with 5 μM BODIPY TR-labeled ceramide-BSA (Invitrogen, Carlsbad, CA) for 30 minutes at 4°C. Cell were then rinsed with cold medium and were incubated for another 30 minutes at 37°C. Cells were then washed and stained with Hoechst 33258 (Sigma-Aldrich, St. Louis, MO, USA). This was followed by several washes and cells were examined and imaged with a fluorescent Zeiss Axiovert 200 M microscope, and AxioVision software.

### Histopathology and immunohistochemistry

Cells were fixed in 1% paraformaldehyde (v/v) for 25 minutes, and then washed in PBS. Ceramide labelling was conducted using an antibody from Alexis (San Diego, CA. USA). Cells that were stained for UGT8 (antibody was obtained from Abcam, Cambridge, MA. USA) were permeabilized and then the antigenic sites were un-masked using sodium citrate buffer. Immunostaining was then done using a Histostain-plus kit (Invitrogen, Carlsbad, CA) following the manufacturer’s instructions, previously described [3, 6]. Images were acquired using LEICA DM LB light microscope and Leica IM1000 software.

Tumour tissues were sliced into two halves. One half was embedded in optimal cutting temperature (OCT) media, and stored at -80 C. Cryo- 8μm sections were prepared, and used for ceramide labelling as described above. The other half was fixed in freshly prepared 1% paraformaldehyde (v/v). Histopathology was assessed using hematoxylin and eosin (H&E) staining.

Tumour cell death was evaluated using a TUNEL assay and fibrosis was revealed using Masson trichrome staining. TUNEL assay results were evaluated using Image J as was previously described [6, 8], where areas of cell death were quantified relative to the total area of tumour sections.

### Clonogenic assays

After treatments, 10^3^ cells were plated in triplicates in 35mm tissue culture dishes from each treatment condition. Cells were then incubated at 37°C and 5% CO2 for up to a week. The developed colonies were then fixed and stained with 0.3% methylene blue/methanol for 20 min. The numbers of the colonies were counted and compared. Mann-Whitney was used to determine statistical significance.

### Immunocytochemistry and electron microscopy imaging

Cells were fixed in 2% paraformaldehyde/glutaraldehyde (v/v), followed by incubation in 50 mM glycine. Permeabilization was performed with 0.1% triton in 0.1% sodium citrate followed by incubation in 50 mM glycine. Cells were then washed with PBS, and incubated with either UGT8 antibody (1:50), or ceramide antibody (1:10), or with both (for double labelling), overnight at 4°C. After three washes with PBS, cells were incubated with either or both secondary antibodies (1:50 dilution): goat anti-rabbit (10nm Gold; ab27234), donkey anti-mouse (12nm Gold; ab105277) at room temperature for 45 minutes; both were obtained from Abcam (Cambridge, MA. USA). To enhance the labeling 0.5% gold(III) chloride was used for 10 minutes at 4°C, after which cells were washed and post fixed in 1% glutaraldehyde for 10 minutes, and stained with 2% uranyl acetate for 5 minutes. Washes with water followed and cells were embedded in GACH (Glutaraldehyde/Carbohydrazide), a lipid-retaining embedding polymer, according to the manufacturer’s instructions. Cells were dehydrated gradually in 20% GACH + 80% H20 (overnight at 4°C), then 50%, 80% GACH for 2 hours each at 4°C, and 100% GACH for 1 hour at 4°C. Cells were then embedded in fresh GACH and were incubated at 37°C for 14 hours to polymerize. Samples were then cemented to empty epoxy blocks, trimmed and sectioned. Both uranyl acetate and GACH were obtained from electron microscopy sciences (Hatfield, PA, USA). JEOL 1011, transmission electron microscope, was used to obtain the images, which were acquired at 50,000X magnification. Chemicals and materials were purchased from Electron Microscopy Sciences (Hatfield, PA, USA).

### In vitro treatments

PC3 cells were collected and treated in suspension. As previously described [3, 21], approximately 3X10^6^ cells were used for each treatment condition. Ultrasound-stimulated microbubble treatments were conducted using a concentration of 3.3% (v/v) Definity microbubbles (Lantheus Medical Imaging Inc., Mississauga, ON, Canada). Approximately 6x10^8^ microbubbles were added to cell suspensions, which were then exposed to ultrasound pulses with a peak negative pressure of 240 kPa and a frequency of 500 kHz. Cells were treated with a duty cycle of 10% for 30 seconds, and were continuously stirred to ensure efficient exposure to ultrasound waves. Treatment conditions included: MB+US alone, XRT (8Gy) alone, or combined MB+US+8Gy treatment, where XRT was administered soon after ultrasound-stimulated microbubble exposure. Irradiation was carried out at 160 kVp energy with a dose rate of 200 cGy/minute. After treatments, cells were incubated at 37°C for up to three hours. Approximately 10^6^ cells were collected and stored at -80 for RNA analysis. The remaining cells were used for clonogenic (colony) and immunolabelling assays.

### In vivo treatments

Stably transfected PC3 cells were used to generate xenograft tumours in six week old CB-17 sever compromised immune-deficient (SCID) mice, obtained from Charles River (Sherbrooke, Senneville, Canada). Tumours were generated by subcutaneous injection of 10^6^ cells/50μL in the hind leg of each mouse. Tumours were treated after reaching 7–10 mm in diameter. Three to five mice were used per condition per tumour strain (wild type, sham, *UGT8* down-regulated, and *UGT8* up-regulated). Mice were anaesthetized prior to treatments with a mixture of xylazine (10 mg/kg), and ketamine (150 mg/kg). Treatment conditions included: MB+US, or 8Gy, or MB+US+8Gy. As previously described [2, 6–9, 11], a concentration of 3% (v/v, in reference to mouse blood volume) microbubbles was used. The microbubbles were formed by shaking at 3000 rpm for 45 seconds. The therapy system is made of an amplifier (Ritec, RPR 4000), a wave generator (Tektronix, AWG520), an ultrasound transducer (IL0509HP, Valpey-Fisher, MA. USA), and an acquisition system (Acquiris CC103) to adjust the focus of the transducer. A total of 750 millisecond of exposure to ultrasound using a frequency of 500 kHz with 16 burst cycles for 5 minutes, with an overall duty cycle of 0.25% was implemented to prevent vasculature collapse, and to allow for blood vessel refill. For the combined treatments, tumours were irradiated soon after the MB+US treatments using a Faxitron X-ray cabinet at a rate of 200 cGy/min. Tumour samples were collected 24 hours after treatments, with a sliced half of a sample fixed in formalin and the other sliced half embedded in OCT medium, and stored in -80 freezer for future immunolabelling processing. Conditions used were based on physical parameters identified in previous studies (8, 9)

### Ethics statement

*In vivo* experiments were carried out after the approval of the animal care committee at Sunnybrook Health Science Centre (Comparative Research), under animal use protocol # 12–425. To eliminate any animal suffering, mice were anesthetized with 2% Isoflurane in O2 flow of 1 L/h, during injections. After tumours reaches 7 mm; animals were anaesthetized with 80–100 μL of a mixture of 1mg/kg Acepromazine, 5 mg/kg Xylazine, and 100 mg/kg Ketamine or alternatively with 2% Isoflurane in O2 flow of 1 L/h to eliminate any potential pain during treatments or imaging. Animals were sacrificed using an anesthetic overdose and according to the standard operating procedures stated by comparative research.

### Power doppler and photoacoustic imaging

Mice were sedated before imaging as was described above, and vascular indices were measured by calculating the change from power Doppler images acquired before treatments and 24 hours later. The power Doppler data were obtained using VEVO-770 (FUJIFILM VisualSonics, Toronto, Canada), and a RMV-710B transducer with a central frequency of 25 MHz and with the following B-mode settings: 20-dB gain, a step size of 0.2 mm and a scan speed of 2 mm/s. Acquired images were analyzed using MATLAB-based in house software (MathWorks, Natick, MA) as previously described [2, 11]. Regions of interest included all the frames resulting in typically 20–25 frames per tumour. The resulting averaged vascular index before treatment was then compared to that after treatment to find the change per tumour and per treatment-type.

Photoacoustic data were also acquired from the same tumours with oxygen saturation and related parameters measured before and twenty four hours after treatment. These data were obtained using Visual Sonics 2100 with a LAZR light source (Visual Sonics, Toronto, Canada) and an 18 MHz central frequency transducer (LZ-250). B-Mode images were acquired at a gain of 20 dB, and a step size of 0.15 mm. The photoacoustic signal was obtained using a gain of 51 dB. The acquired photoacoustic images resulted from using two optical wavelengths, 750 nm and 850 nm, to determine deoxygenated and oxygenated hemoglobin. Visual Sonics 2100 software was used to analyze the data and to determine the level of oxygen saturation. Regions of interest were selected as was described for the power Doppler analyses, excluding the skin. Data from every other frame were collected and the calculated oxygen saturation levels were then averaged and compared. This amounted to 10–15 frames per tumour on average. Data from before and after treatment were then compared to determine the change per tumour per condition.

### Statistical analyses

Data were statistically evaluated using either a t-test or Mann Whitney test with a 95% confidence level, where *P < 0*.*05* was considered significantly different. Additionally, both One-way ANOVA and Kruskal Wallis (Nonparametric One-way ANOVA) were performed and included in the supplementary results (S1 Table). All the statistical analyses were carried out using GraphPad Prism 4 (from GraphPad Software, Inc. La Jolla, CA, USA).

## Results

### Down-regulating and up-regulating UGT8: Effect on ceramide

**Fig 1:**
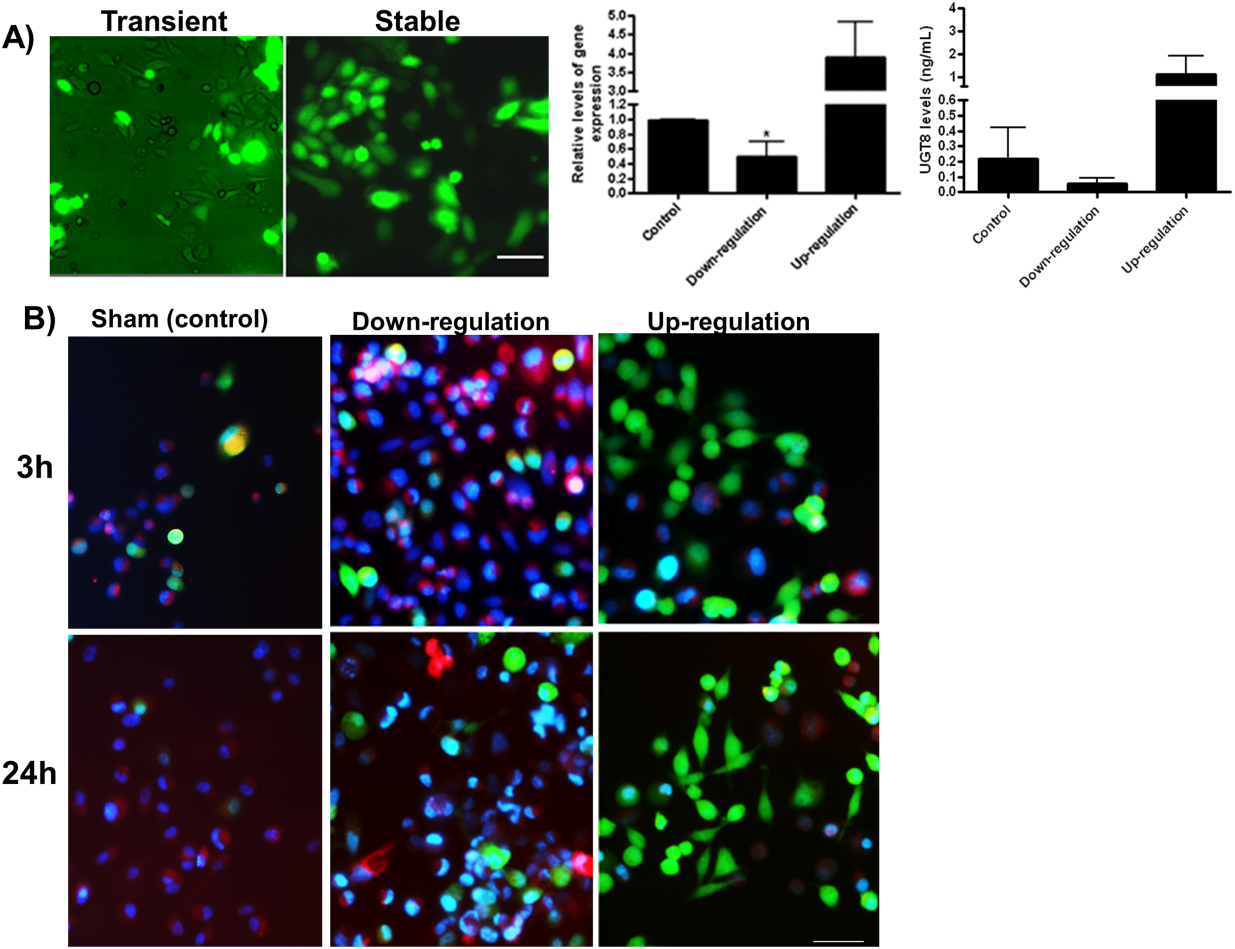
Transfection of PC3 cells *in vitro* and ceramide trafficking. Transfected cells were identified by the fluorescence of GFP. A) Transient transfection, showing fewer transfected cells than in the stably transfected cells. The levels of the *UGT8* gene and the UGT8 protein were evaluated after the stable transfection using RT-PCR, and ELISA, which verified the change in the levels of regulation. B) Ceramide trafficking in live images of cultured PC3 cells using BODIPY TR-labeled C_5_ ceramide (red fluorescence), nuclear position is indicated by the nucleic acid stain Hoechst 33258 (blue fluorescence) with green fluorescence indicating transfection of constructs. After 3 h, control and down regulated cells demonstrtaed a higher level of ceramide uptake (red) that masks the green fluorescence compared to the cells over expressing UGT8. After 24 h, control and down regulated cells retained the exogenous ceramide (red) contrary to the cells over expressing *UGT8*. Scale bars represent 50 μm.

A shRNA construct against UGT8 that down-regulated the gene by 50% was used for experiments. This level of loss of function was selected in order to avoid potentially high levels of cyto-toxicity. Transient transfection had a 40–50% efficiency and thus, to obtain detectable effects and enable investigation over a period of time, a stably transfected PC3 cell line was generated. Controls included cells stably transfected with an open reading frame (ORF) construct for *UGT8* over-expression, and cells stably transfected with scrambled DNA. *UGT8* down-regulation lowered expression levels to 0.5 +/- 0.2 times relative to control cells, and up-regulation raised gene levels to 3.9 +/- 0.9 fold more than control cells. Consequently, UGT8 protein levels were also changed as was observed when carrying out an ELISA assay. UGT8 activity in cells was verified using a fluorescently labeled short analog of ceramide (BODIPY TR-labeled C5-BSA) added to controls, down-regulated and up-regulated UGT8 containing cells (Fig 1). Three hours after incubation, the fluorescent C5 was up taken by all cells, however, an increased retention of the labeled ceramide was observed in cells where UGT8 was down-regulated (red staining) unlike the levels in the control or in the cells over expressing UGT8 (more green and less red). Cells were also examined 24 hours later where UGT8 had further metabolized the ceramide analog causing a shift to green fluorescence.

### UGT8 attenuation effects on treatment conditions and cellular survival

**Fig 2:**
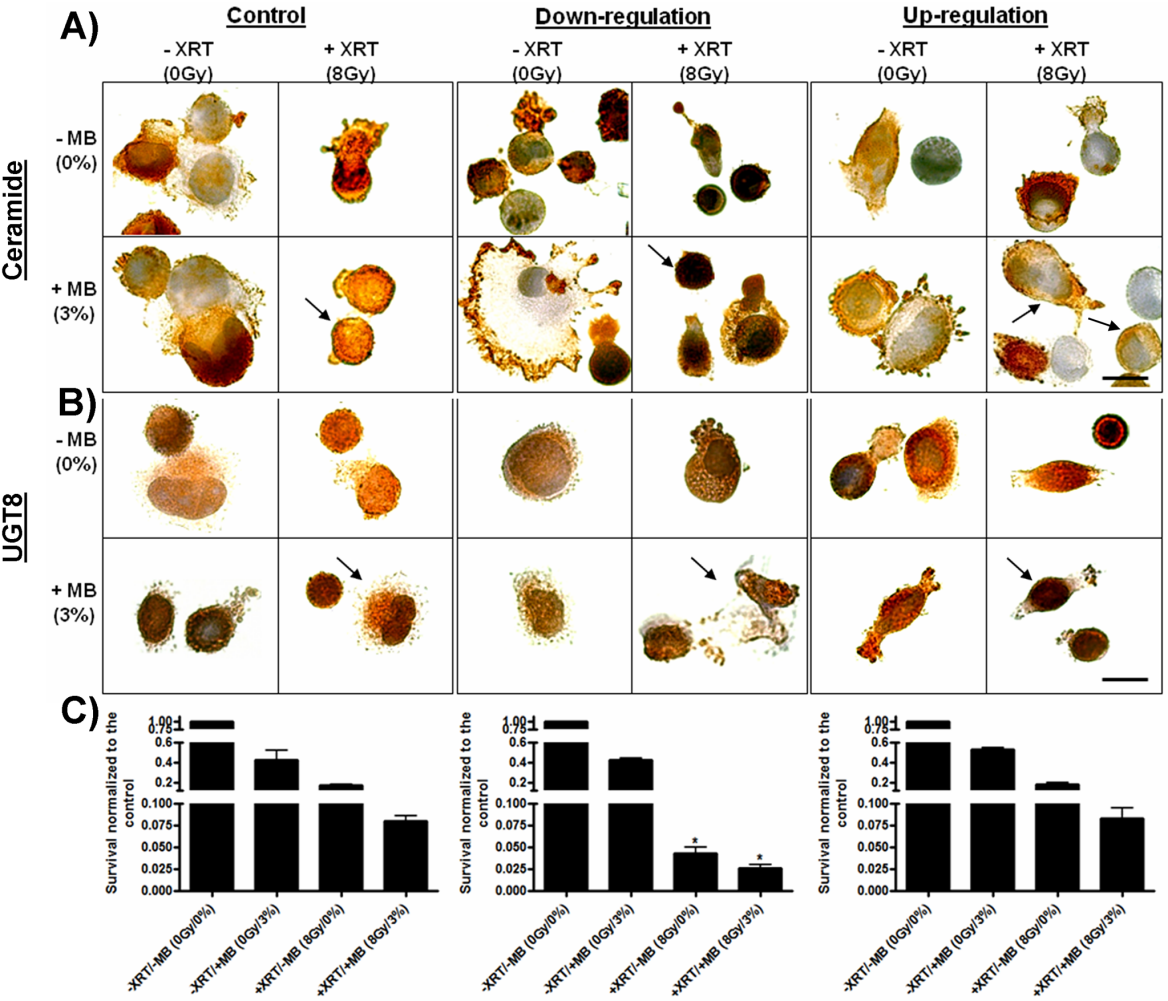
Ceramide, UGT8 labelling, and cellular survival after treatments. A) Control cells demonstrating increased levels of ceramide stained under various treatment conditions (brown, arrow). More condensed brown labelling was observed in down regulated cells under various treatments (arrow). In contrast, cells with up regulated UGT8, that were also subjected to similar conditions, demonstrate less ceramide labeling (arrows). B) UGT8 labelling was most intense in the up-regulated cells compared to the control or to the down-regulated cells (arrows). Scale bar represents 25 μm. C) Clonogenic assays indicate that control and up-regulated cells were less susceptible to treatment when compared to cells stably transfected with *UGT8* inhibitory shRNA.

PC3 cells *in vitro* stably expressing down-regulated, wild-type levels, and up-regulated UGT8 were treated with either US+MB alone, XRT alone or a combination of both. After treatments cells were fixed and immuno-labeled either for ceramide or for UGT8 (Fig 2A & 2B). Results indicated increased ceramide levels in wild-type cells subjected to either treatment with either US+MB, XRT, or the combination of the two treatments (Fig 2A). More elevations were observed in cells with down-regulated *UGT8* indicating greater amounts of ceramide in response to all three treatments. In contrast, cells with up-regulated *UGT8* indicated lesser amounts of ceramide in response to treatments as expected. Immuno-labeling of UGT8 confirmed similar effects with ultrasound-stimulated microbubble treatments in wild type (control) cells causing an up-regulation of UGT8 in response to cell stress. Down-regulation caused decreased UGT8 levels for treatment conditions compared to wild-type (control) cells. In contrast, up-regulation of UGT8 caused more immunolabelling especially with treated samples with the greatest response apparent in combined treatments of US+MB+XRT (Fig 2B). Electron microscopy indicated a co-localization of UGT8 and ceramide at the Golgi and endoplasmic reticulum (supplementary data, S1 Fig).

Cell survival was investigated using clonogenic assays. Results indicated a significant enhancement of cells to radiation with the use of US+MB with additive effects *in vitro* when US+MB were combined with XRT. Treatments with US+MB resulted in 0.4 +/- 0.1 survival compared to control, XRT treatment resulted in 0.2+/-0.003 cell survival, and the combination resulted in 0.08 +/- 0.006 survivals. The amount of cell death increased and was apparent further when UGT8 was down-regulated, *P* < 0.05 (Fig 2C) with the combination treatment now resulting in 0.03 +/- 0.003 survival compared to control cells. Up-regulation of UGT8 resulted in a relative protection of cells from the effects of US+MB+XRT (0.8 +/- 0.01 compared to control) and also for treatments with US+MB alone 0.5 +/- 0.02 compared to control) (supplementary data, S2 Fig). Although the survival of treated cells was reduced as expected in the three groups (control, up-regulated and down-regulated UGT8), cells stably transfected with *UGT8* inhibitory shRNA were even more susceptible to treatments when compared to the other two groups, where survival was further reduced and the treatment difference from the other groups was significant, *P* < 0.05 (Mann-Whitney test). Data from all the groups were compared and were found to be statistically different *P* < 0.0001 (One-way ANOVA, S1 Table).

### In vivo analyses of UGT8 effects on tumour responses to treatment

**Fig 3:**
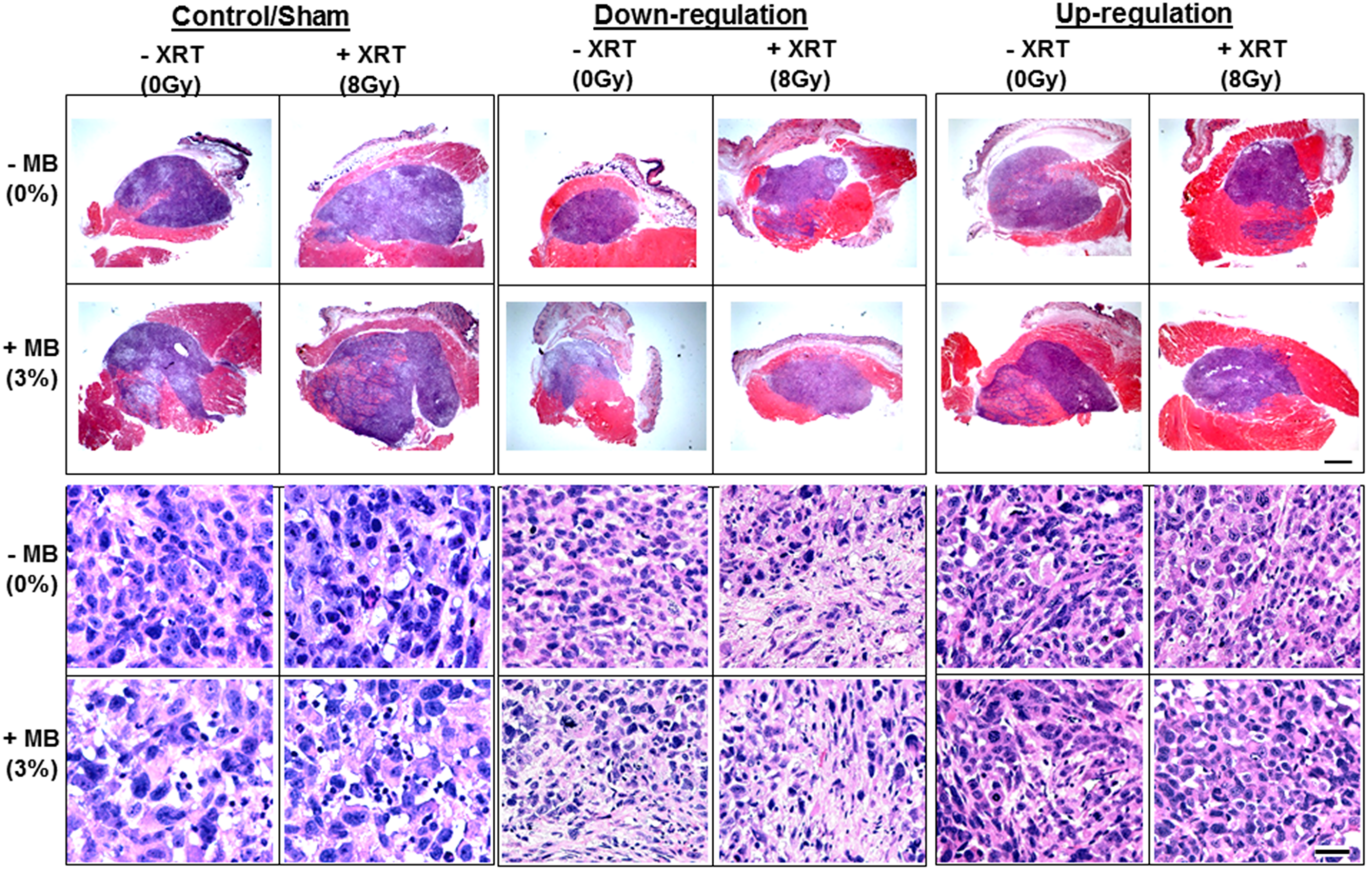
H & E stained sections from xenograft PC3 modified tumours not treated with combinations of MB/XRT. The upper two panels show low magnification whole tumour sections and the lower two panels illustrate high magnification images. Controls, not transfected, or transfected with scrambled shRNA (sham) demonstrated apoptotic cell death more with combined treatments and less with single treatments. However, tumours induced by cells stably transfected with anti-*UGT8* sh-RNA, show different types of cell death and fibrosis. Cell death was less obvious in xenograft tumours initiated from cells with up-regulated *UGT8*. Scale bar for low magnification images is 1mm and for high magnification represents 50 μm.

In order to investigate the effect of UGT8 up- and down-regulation on tumour tissues, xenograft tumours were generated using stably transfected PC3 cells up- and down-regulated with respect to UGT8 expression. Controls included tumours generated from either wild-type PC3 cells or from PC3 cells stably transfected with scrambled DNA (sham). Animal-borne tumours from each PC3 strain were exposed to no treatment, US+MB microbubble treatments, XRT treatments, or the combination of the two treatments (Fig 3).

**Fig 4:**
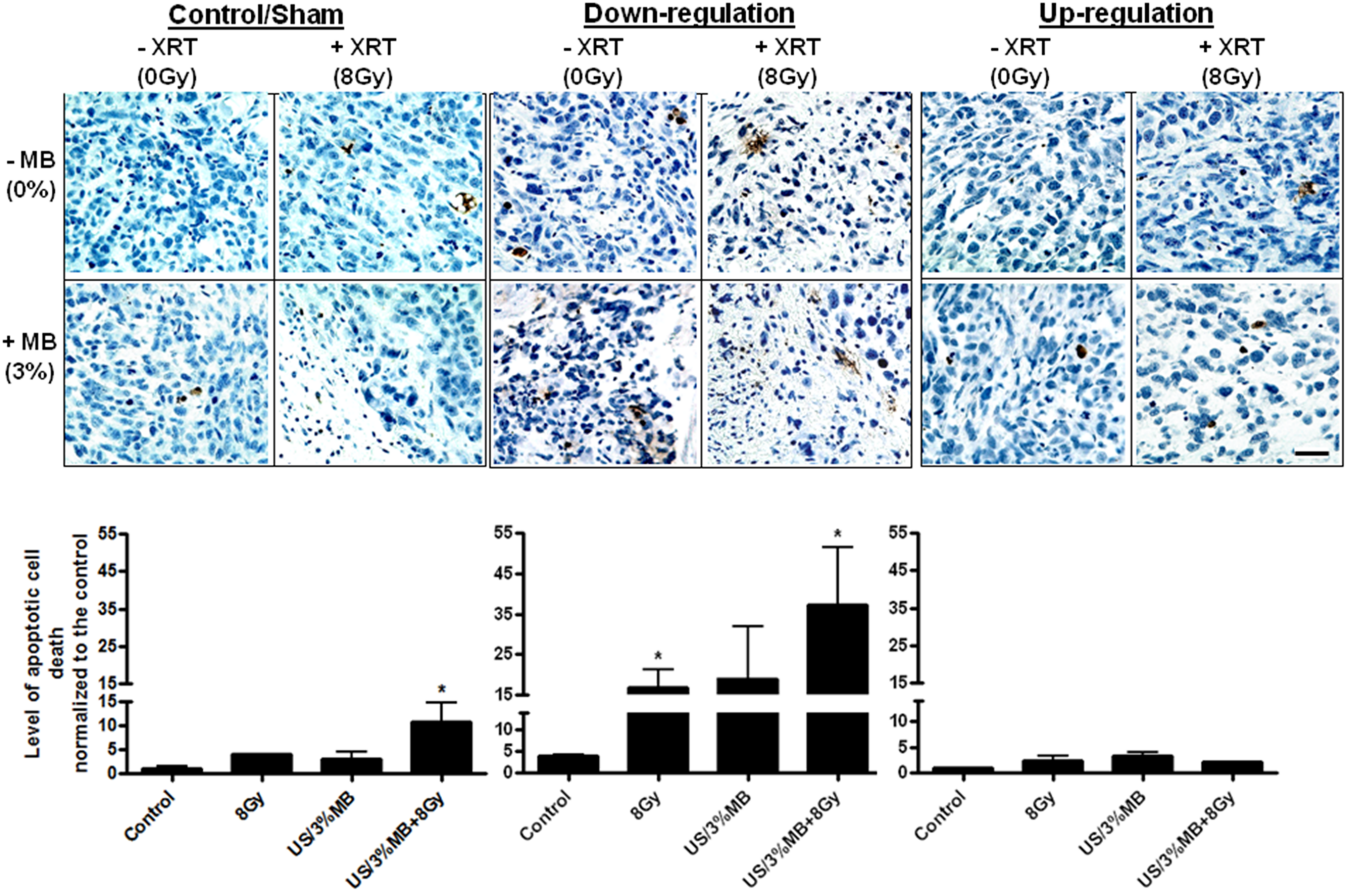
TUNEL staining of xenograft PC3 modified tumours sections treated with various conditions. Control and sham tumours illustrate an increase in levels of apoptotic cell death which was significant in the combined treatments. A further increase in cell death is observed in tumours induced by cells stably transfected with anti-*UGT8* sh-RNA. In contrast, less cell death is observed with up-regulated *UGT8*. Scale bar represents 50 μm.

The histology of regular/sham PC3 tumours illustrated apoptotic cell death and specifically more with the combined treatment. Tumours where *UGT8* was down-regulated illustrated various types of cell death that included apoptosis, necrosis, as well as fibrosis. There appeared to be even more cell death and tumour destruction with treatments in this tumour type. On the contrary tumours generated with *UGT8* up-regulated PC3 illustrated less cell death overall, potentially because of ceramide depletion by the elevated UGT8 levels. Further evaluation of apoptotic cell death was carried out using TUNEL assay in control, *UGT8* up-regulated, and *UGT8* down-regulated tumours (Fig 4). Results revealed a significant increase in cell death when comparing the combined treatments in the sham group (*P* < 0.05) or in the down-regulated group (*P* < 0.018) to their perspective controls. Radiation treatments alone in the down regulated group also significantly increased cell death (*P* < 0.018). However, such significant changes were not observed in the *UGT8* up-regulated group. Further, when comparing cell death between the groups, specifically for the combined treatments, cell death was significantly higher in the *UGT8* down-regulated tumours when compared to either that of the control/sham (*P* < 0.018), or to the *UGT8* up-regulated (*P* < 0.018) tumours. Cell death with the combined treatment depressed from 10.7 +/- 4 in the control/sham group to 2 +/- 0.04 in the *UGT8* up-regulated group and was increased to 37 +/- 14 in the *UGT8* down-regulated group. Data from all the groups were compared and were found to be statistically different *P* < 0.0282 (One-way ANOVA, S1 Table).

**Fig 5:**
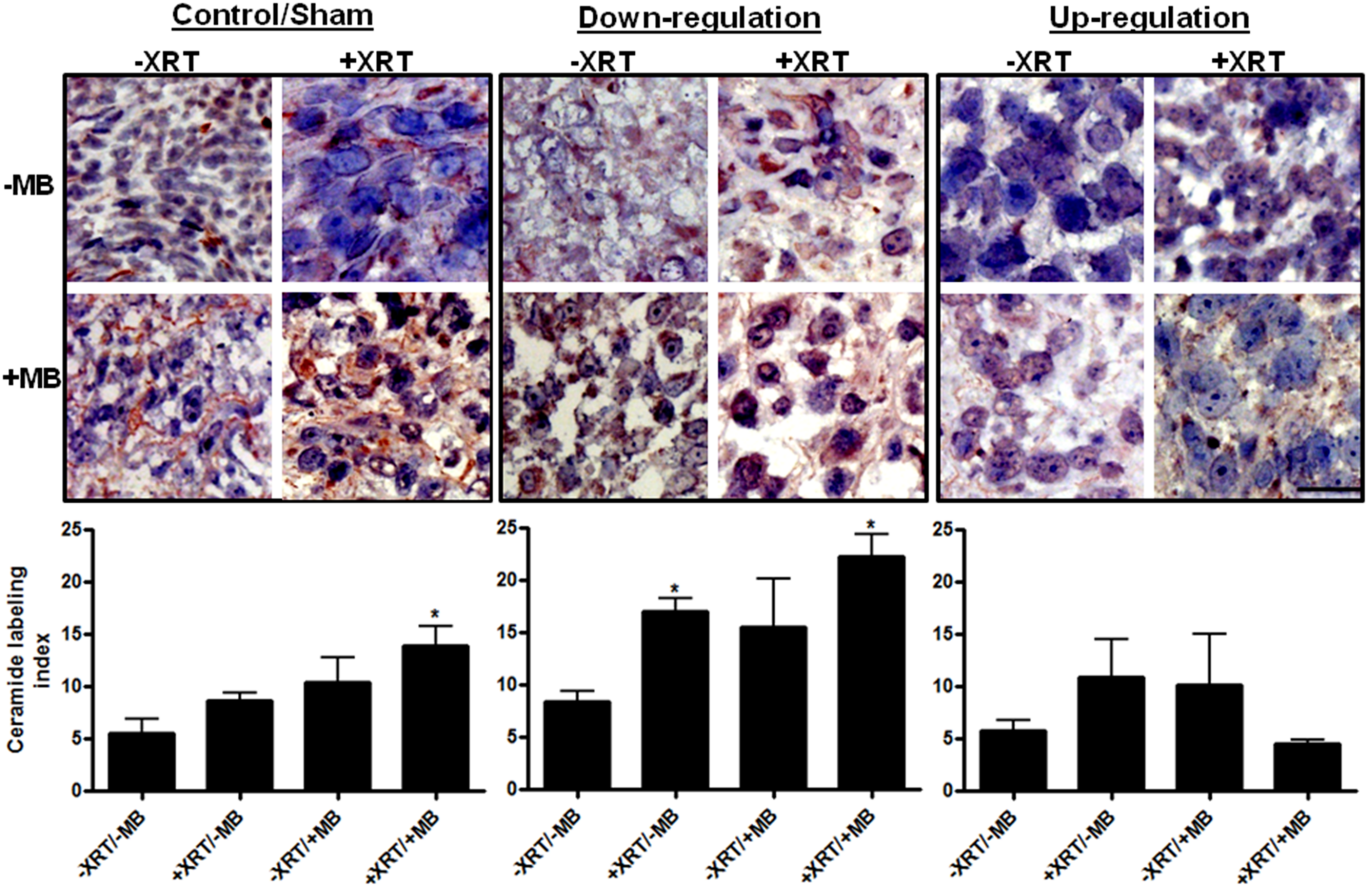
Ceramide labelling of xenograft PC3 modified tumours sections treated with various conditions. Control and sham tumours illustrate an increase in levels of ceramide which is significant in the combined treatments (dark red). An increase in ceramide is observed in tumours induced by cells stably transfected with anti-*UGT8* sh-RNA. Less ceramide is observed with up-regulated *UGT8*. Magnification bar represents 20 μm.

Ceramide immunolabelling was carried out on the control/sham, *UGT8* up-regulated and *UGT8* down-regulated tumours (Fig 5). Results in control tumours indicated increased ceramide production with XRT treatment, exposure to US+MB, and maximally in tumours exposed to the combination of XRT and US+MB (*P* = 0.0182). In the *UGT8* down-regulated tumours there was an overall increased level of ceramide compared to the control tumours. The *UGT8* up-regulated tumours exhibited a decreased level of ceramide production relative to the control tumours. The ceramide labelling index for the combined therapy decreased from 14 +/- 1.8 in the control/sham group to 4.5 +/- 0.4 in the *UGT8* up-regulated group and was increased to 22 +/- 2 in the *UGT8* down-regulated group (*P* = 0.0035). Data from all the groups were compared and were found to be statistically different *P* < 0.0001 (One-way ANOVA, S1 Table).

**Fig 6:**
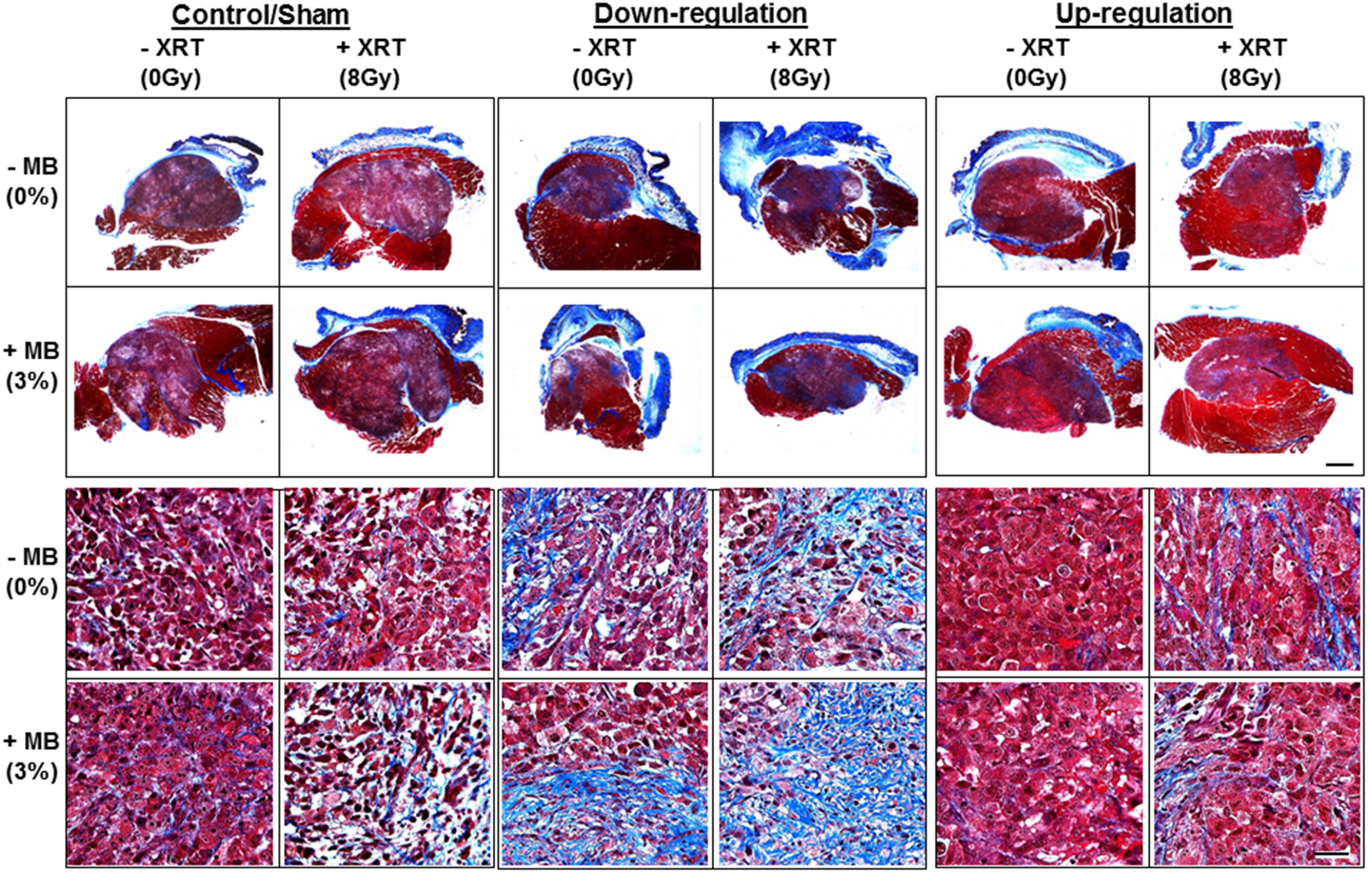
Trichrome staining of xenograft PC3 modified tumours sections. Whole tumour sections and high magnification images show collagen fibers (stained blue), cytoplasm and muscles are red, and nuclei are black. More blue in the down regulated tumours (visible under higher magnification) indicates more cell death replaced by fibrotic tissues. Less blue in the up-regulated samples indicates less cell death resulting in less fibrosis. Scale bar for low magnification images is 1mm and for high magnification represents 50 μm.

Fibrosis in tumours, which is a post cell death process, was also evaluated using Masson Trichrome staining and revealed treatment effects (Fig 6). In control tumours there was increasing fibrotic change with radiation treatment (blue staining), further fibrosis in comparison with ultrasound-stimulated microbubble exposure, and the most evident in the combined treatment of ultrasound-stimulated microbubble exposure and radiation treatment. In *UGT8* down-regulated tumours there was more overall fibrosis evident in Masson stained samples (increased blue staining). In contrast, in the *UGT8* up-regulated samples there was less overall fibrosis in comparison to control samples (less prominent blue staining and overall more red staining).

### Treatment effects on tumour blood flow and oxygen saturation

**Fig 7:**
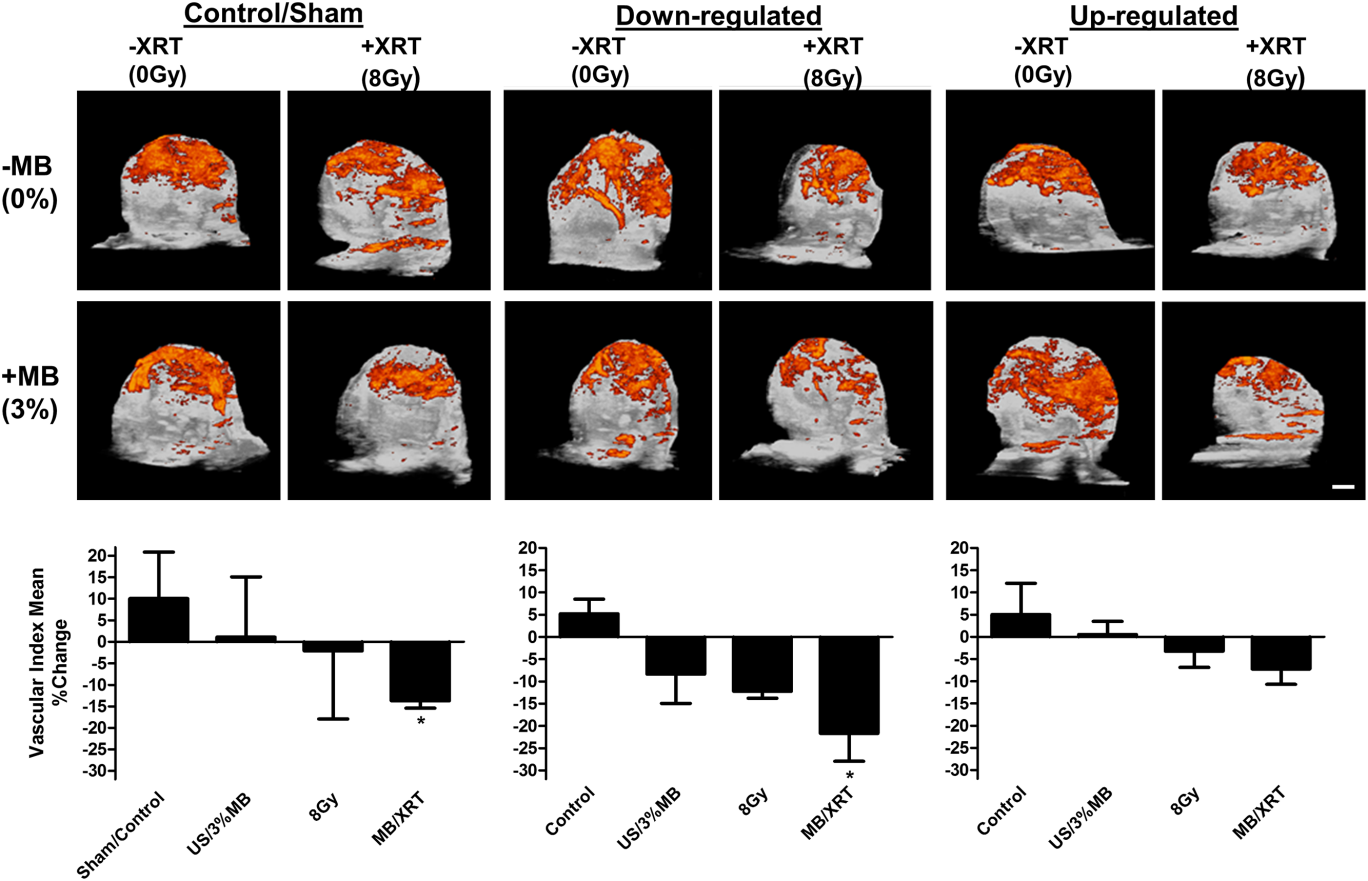
Ultrasound images of xenograft PC3 modified tumours in vivo and power Doppler analyses post-treatment. Vascular index change in vasculature 24 hours after treatments is significantly different with the combined treatments of either sham or down regulated tumours, where a reduction in the vascular index is observed, with more reduction in the down regulated xenograft. Although there is a reduction in the vasculature of the up-regulated tumours as well, it is not statistically significant. Scale bar represents 1 mm.

Given the known effects of radiation and ultrasound-stimulated microbubbles on tumour vasculature, real time investigation of the change in tumour vasculature was conducted using power Doppler imaging of the tumours before and 24 hours after treatments. Data analyses indicated a reduction in the vascular index in the combined treatments in all the groups. This was only significant in the control animal group (*P* < 0.05) and in the *UGT8* down-regulated group (*P* < 0.029) when compared to their prospective controls (Fig 7). Although the vascular index was also reduced in the up-regulated group as well, it was not significantly different from the control. The vascular labelling index for the combined therapy increased from -13.7 +/- 1.7 in the control group to -7.3 +/- 3.4 in the *UGT8* up-regulated group and was depressed to -21.7 +/- 6.3 in the *UGT8* down-regulated group. Data from the controls and the combined treatments from all the groups were compared and were found to be statistically different *P* < 0.0133 (One-way ANOVA, S1 Table).

**Fig 8:**
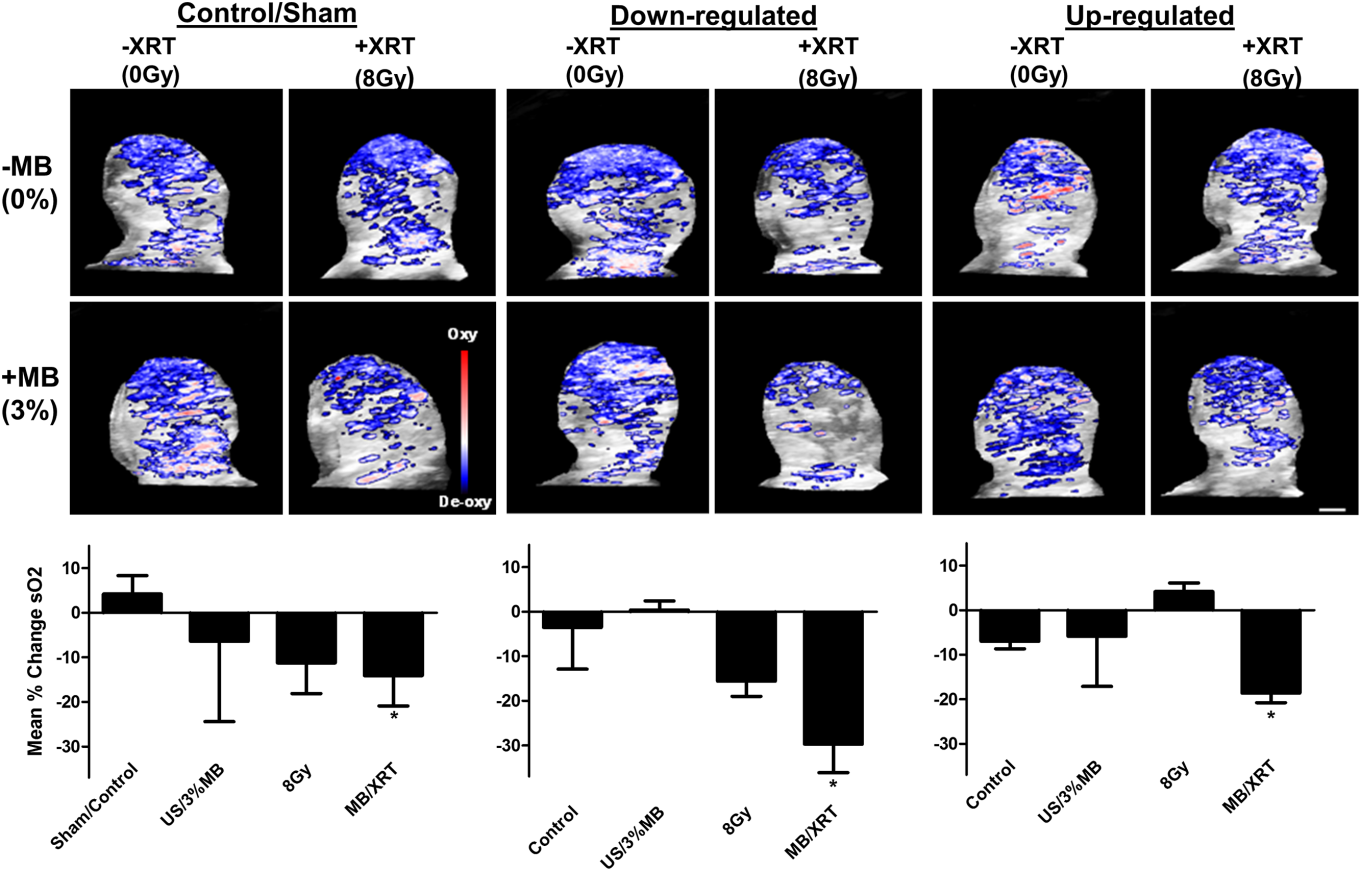
Photoacoustic post-treatment images of xenograft PC3 modified tumours in vivo and oxygen saturation analyses. Percent change in oxygen saturation levels twenty-four hours after treatments is significantly different with the combined treatments, but not with the single treatments of sham, down regulated and up regulated tumours, where a reduction in the saturation is observed, with more reduction in the down regulated xenograft. Scale bar represents 1 mm.

Photoacoustic imaging was also used to investigate anti-vascular treatment effects. This was accomplished through the evaluation of oxygen saturation levels that can be acquired from photoacoustic imaging, a hybrid modality that combines optical and ultrasound imaging. The use of the 750 nm wavelength reflected the levels of deoxygenated hemoglobin, where the use of an 850 nm wavelength reflected the levels of oxygenated hemoglobin. The levels of oxygen saturation were significantly reduced in the combined ultrasound-stimulated microbubble and radiation treatments with *P* < 0.046 (control), *P* < 0.047 (down-regulated) and *P* < 0.016 (up-regulated) (Fig 8). In the *UGT8* down-regulated tumours greater levels of hypoxia were observed whereas in the *UGT8* up-regulated tumours lesser levels of hypoxia were observed. The oxygen saturation index for the combined therapy changed from -14 +/- 6.8 in the control group to -18 +/- 2 in the *UGT8* up-regulated group and then relatively decreased to -29.7 +/- 6.4 in the *UGT8* down-regulated group. Data from the controls and the combined treatments from all the groups were compared and were found to be statistically different *P* < 0.0243 (One-way ANOVA, S1 Table).

**Fig 9:**
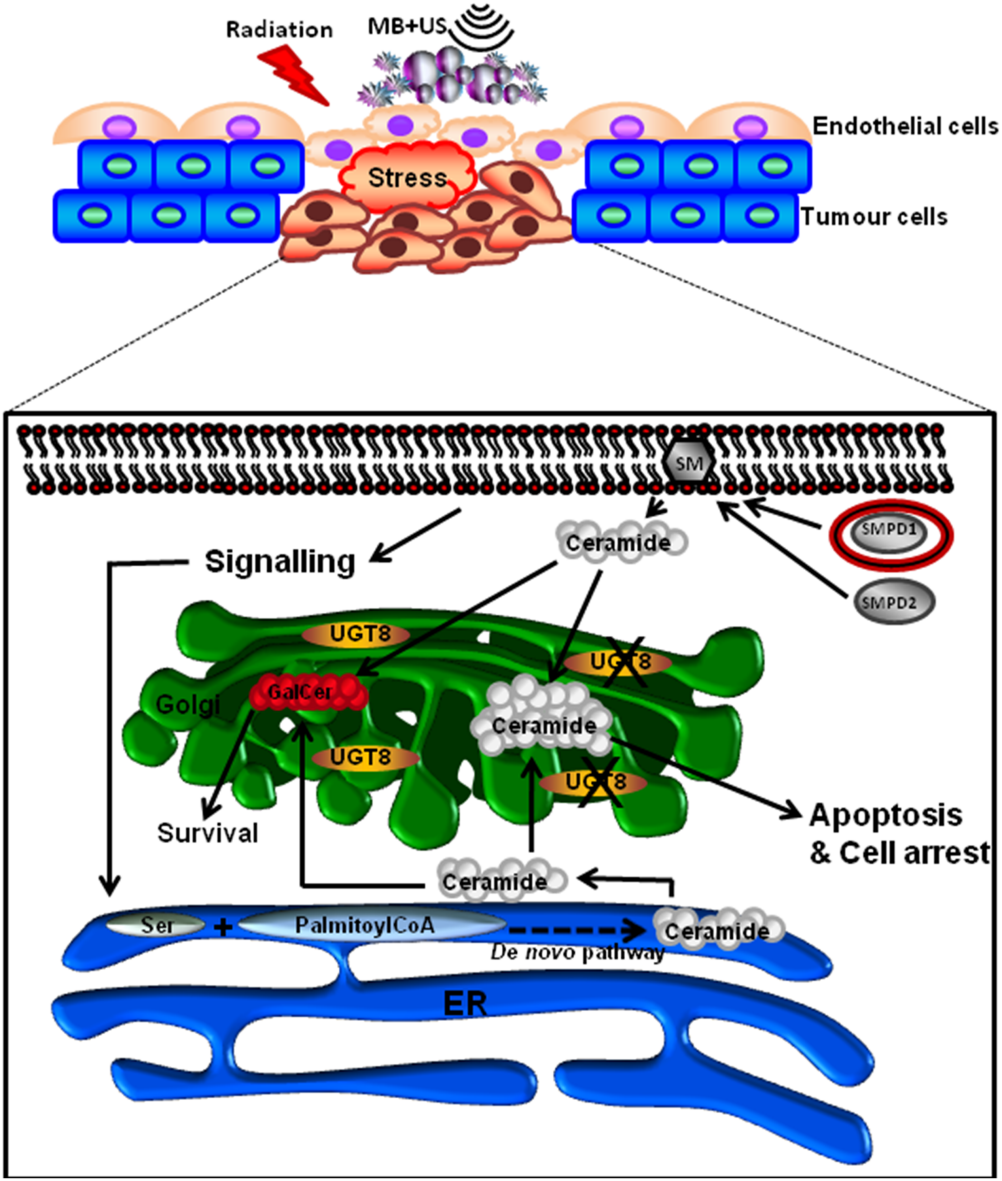
Summary of UGT8 signalling and its effect on ceramide metabolism. When UGT8 levels are reduced, ceramide, either produced at the cellular membrane or in the endoplasmic reticulum, accumulates or initiates an apoptotic signal. However, when UGT8 levels are elevated, it adds a sugar moiety to ceramide in the Golgi apparatus, converting it to galactosylceramide, and this leads to the reduction of ceramide levels and possibly its apoptotic signalling, thus making cells more resistant to therapy.

A summary of the investigated UGT8 signalling was illustrated in the schematic Fig 9, which indicates that UGT8 levels are linked to ceramide metabolism to achieve homeostasis in signaling.

## Discussion

It has been previously demonstrated that exposure of tumour vasculature to ultrasound-stimulated microbubbles can enhance effects of radiation significantly. In that work, *in vivo* experiments in which ultrasound-stimulated microbubble and radiation treatments were conducted demonstrated a synergy dependent on blood vessel disruption [2, 6, 8, 9, 12–13]. Previous data indicated that tumour cell death is secondary to vascular disruption resulting from endothelial cell death. The mechanism by which endothelial cell damage results in tumour cell death and the role, if any, of ceramide signalling in modulating cell death in tumours remained to be investigated.

Previous work has focussed on the role of the endothelium in initiating a radiation-enhancing effect in response to treatment exposure. Despite the initial damage being caused in the vasculature there are ceramide effects and stressors on tumour cells well beyond the vasculature as a results of initial blood vessel damage. The work here focussed on the mechanism of damage transduction into tumour parenchyma, an event separate from endothelial damage but linked to the anoxia caused and ceramide signalling. Specifically, knowing membrane related damage and lipid-based signalling were important components involved in radiation-enhancement the study here investigated the role of lipid metabolism and membrane repair in this effect. Here experiments focused only on modifying the tumour cells making them up- or down-regulated in UGT8 which can affect lipid-based cell membrane repair. Furthermore, the spatial association of vascular disruption, ceramide production, apoptosis and fibrosis will be studied elsewhere as the work here only provides static results from one experimental time.

In this study UGT8 gene expression was modulated to test the importance of ceramide signalling in transducing the effects of blood vessels disruption into tumour cells. UGT8 is an enzyme, which is involved in ceramide metabolism and its levels were found to be elevated in a number of cancers [22–24]. The up-regulation of this enzyme resulted as expected in lower intracellular ceramide levels and its down-regulation resulted in higher intracellular ceramide levels both *in vitro* and *in vivo*. This implicated ceramide as a tumour-cell based factor in responses to vascular disruption as induced by ultrasound-stimulated microbubble exposure combined with radiation-induced cell death, the precise interactions and signalling mechanisms are described in Fig 9.

The down-regulation of *UGT8* resulted in lowering PC3 cellular survival and enhanced radiation effect alone or when combined with ultrasound-stimulated microbubbles *in vitro* and *in vivo*, and the effect was reversed when the gene was up-regulated. The reduced level of survival was also associated with retention or accumulation of ceramide as was demonstrated *in vitro*. A possible interpretation of the results is that when cells are subjected to external stressors such as ionizing radiation, or mechanical disruption of the membranes by ultrasound-stimulated microbubbles, increased levels of ceramide will result in signaling for apoptosis as was previously reported [29–35]. On the other hand, the increased levels of ceramide can also initiate a homeostatic signal to attenuate ceramide levels as a survival mechanism or as a therapy resistance approach, as was previously reported [34, 36, 37]. This can possibly be achieved by converting ceramide to a different molecule, such as galactosyl-ceramide, which is catalyzed by UGT8 enzyme. This can also explain why *UGT8* levels were elevated after treatments with microbubbles and radiation therapy which was previously described [3].

In other work, disruption of ceramide glycosylation was reported to play a role in restoring the apoptotic signal that was lost due to mutated p53, where the resulting increased levels of ceramide enhanced p53-dependent apoptosis [38, 39]. Additionally, the silencing of glucosylceramide synthase (GCS) decreased glycoshingolipids levels and increased ceramide leading to an enhanced apoptotic signal in tumours [40].

In lieu of GSC signaling, it may be possible that UGT8 has a similar effect on ceramide signaling. Here it is proposed that UGT8 acts to assist in the attenuation of ceramide given that results here indicated that the down regulation of *UGT8* resulted in increased ceramide levels *in vitro*. To test this hypothesis, an exogenous fluorescent ceramide was used to track ceramide metabolism and trafficking in live cells stably transfected for either down-regulation or up-regulation. This resulted in an increased retention of ceramide when *UGT8* expression was attenuated, which was also associated with more cell death as oppose to *UGT8* up-regulation where cells were healthier and the levels of ceramide were reduced. Fluorescent labelling of sphingolipids was previously used to study their trafficking, uptake, and metabolism, where it was proven to be a powerful tool, specifically in the trafficking of ceramide to the Golgi- apparatus [41, 42]. Furthermore, emerging reports [24, 43, 44] indicated elevated levels of UGT8 in breast cancer and lung metastasis, and have suggested using UGT8 as a biomarker in the cancer prognosis. Nevertheless, no comprehensive molecular analysis of the signaling mechanism of this molecule is available.

The use of radiation, especially in combination with microbubbles, has been demonstrated to affect endothelial cells inducing apoptotic cell death and disrupting tumour vasculature [2, 6, 7, 11], where tumour cell death is believed to be secondary to tumour vascular disruption. In the above work we investigated what transduces the damage from vasculature into tumour cells. The role of ceramide which is produced under cell stress which would be caused by vascular disruption was therefore modulated.

It was also crucial to investigate the damaging effect of the therapy on tumour vasculature under gene manipulations. This was fulfilled using high frequency high-resolution Doppler ultrasound imaging. Power Doppler is a noninvasive rapid imaging modality that aids in assessing blood flow, and has many applications, such as evaluation of normal vascular development, pathological angiogenesis associated with disease conditions, their progression, or treatment effect [45]. Here, the combined treatment caused a significant decrease in the vascular index in both the control and the down-regulated UGT8 group but not in the *UGT8* up- regulated group. Results indicated less resistance when the gene was down-regulated and more resistance to the therapy with the up regulation. Similar resistance to therapy was also observed with the up- regulation in the *in vitro* experiments. Results coincided with histological evaluations of the *in vivo* experiments as well, as evident in the histopathology and fibrosis evaluation results, where down-regulation led to more cell death and more fibrosis.

Although Doppler ultrasound imaging can provide useful information, there are some limitations when using it to study tumour vasculature, such as small blood vessels, which may not be detected and slow flow velocity, which can also influence the detected signal [46]. Photoacoustic is another imaging modality that enables molecular-based imaging of cellular components such as hemoglobin, where molecular conformational changes can be detected to accurately evaluate oxygen saturation. Oxygen saturation is another important parameter that reflects the level of hypoxia in a tumour, which is a characteristic feature in cancer. The use of photoacoustic imaging, a combined optical/ultrasound imaging modality indicated a significant decrease in the level of oxygen saturation specifically with the combined treatment, which indicated increased levels of hypoxia. These evidence including vascular disruption, tumour cell death and fibrosis give more insight into the mechanism of UGT8 action, where its down regulation indicated a role in the apoptotic process through its effect on ceramide metabolism. As expected down-regulation of UGT8 led to more cell death and more hypoxia leading to greater amounts of cell death and consequent fibrosis. This is consistent with previous work indicating severe vascular insult leading to vascular disruption and cell death [2]. However, here the alteration of UGT8 activity in the tumour cells leading to changes in the amount of cell death indicates a broader role for ceramide in this mechanism. Whether the ceramide in tumour cells is created due to radiation exposure or ischemia and reperfusion injury remains unknown.

## Conclusion

Targeted vascular-disrupting therapy combined with targeting localized UGT8 make the approach a good candidate for further exploration and optimization to achieve an effective cancer therapy with minimal adverse effects on healthy tissues. The use of acoustic energy to enhance the efficacy of ionizing radiation that, in turn, would allow the use of an effective lower radiation dose is a novel approach that was recently developed. This new treatment method would spare healthy tissues while targeting cancer cells. Combining ultrasound-stimulated microbubble therapy with specific gene therapy would open the door to a new revolutionized way of treating cancer that should be further explored. To our knowledge, this is the first study to investigate the UGT8 signalling pathway in prostate cancer, where radiation treatment is combined with ultrasound-stimulated microbubble treatment as a therapy enhancing modality.

## Supporting information

S1 Fig Double immuno-labeling of UGT8 and ceramide in stably transfected PC3 cells. Two different secondary antibodies conjugated to different sizes of gold nano-particles (anti-mouse; 12 nm, labels ceramide, red arrows) and (anti-rabbit; 10 nm, labels UGT8, yellow open arrows). More ceramide labeling and less UGT8 labeling is observed in sham and down regulation samples and the opposite is observed in the up-regulation samples. UGT8 labelling was observed in both the Golgi and the endoplasmic reticulum; both are sites for ceramide metabolism.
(TIF)

S2 Fig Images of formed colonies from the different groups indicating survival after various treatments. (TIF)

S1 Table Additional statistical analyses of data from different validation approaches. (XLS)
