# Supplementary figures and images for: Microbubble-based enhancement of radiation effect: Role of cell membrane ceramide metabolism
Source: PLoS One. 2017 Jul 26;12(7):e0181951. doi: 10.1371/journal.pone.0181951 (PMC5528834; doi:10.1371/journal.pone.0181951)

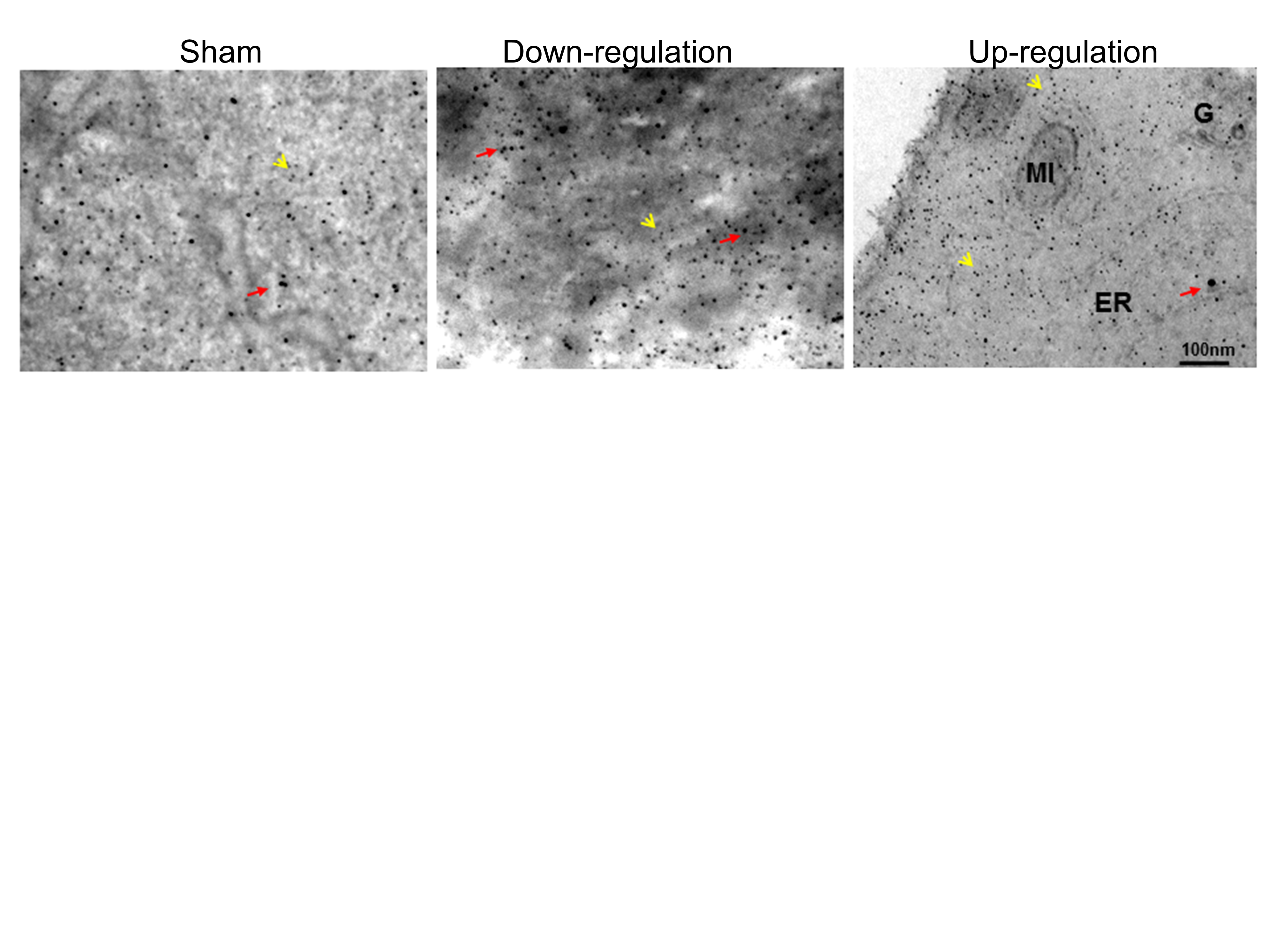

Supplement: S1 Fig — Two different secondary antibodies conjugated to different sizes of gold nano-particles (anti-mouse; 12 nm, labels ceramide, red arrows) and (anti-rabbit; 10 nm, labels UGT8, yellow open arrows). More ceramide labeling and less UGT8 labeling is observed in sham and down regulation samples and the opposite is observed in the up-regulation samples. UGT8 labelling was observed in both the Golgi and the endoplasmic reticulum; both are sites for ceramide metabolism. (TIF) [file pone.0181951.s001.tif]

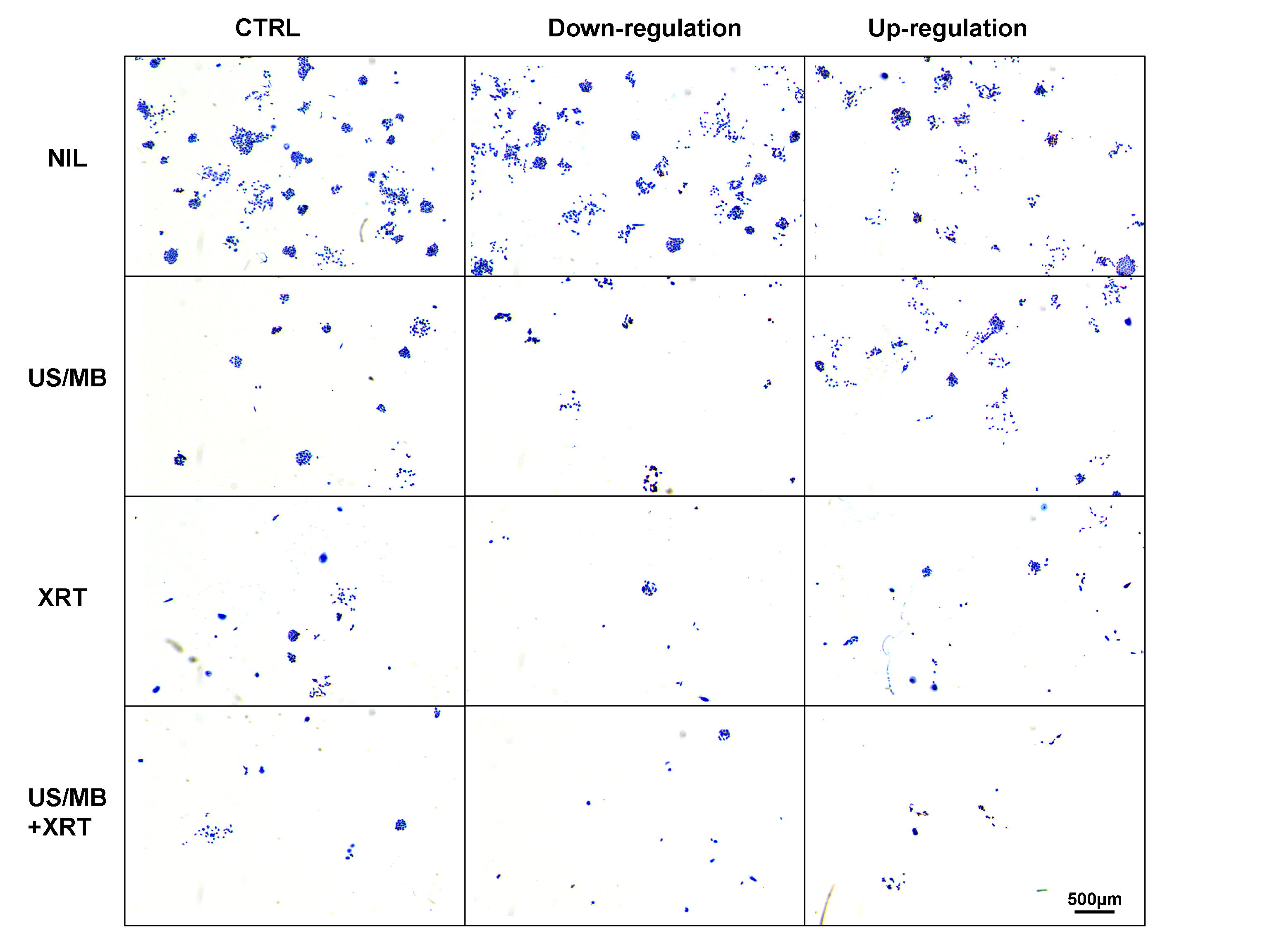

Supplement: S2 Fig — (TIF) [file pone.0181951.s002.tif]
